# Hyponatraemia in imported malaria is common and associated with disease severity

**DOI:** 10.1186/1475-2875-9-140

**Published:** 2010-05-25

**Authors:** Marlies E van Wolfswinkel, Dennis A Hesselink, Robert Zietse, Ewout J Hoorn, Perry JJ van Genderen

**Affiliations:** 1 Department of Internal Medicine, Harbour Hospital and Institute for Tropical Diseases, Rotterdam, The Netherlands; 2 Department of Internal Medicine, Erasmus Medical Centre, Rotterdam, The Netherlands

## Abstract

**Background:**

Hyponatraemia (serum sodium < 135 mmol/L) has long been recognized as a complication of malaria. However, few studies have been done in non-immune adult populations. It has not been investigated previously how hyponatraemia is distributed among the various *Plasmodium *species, and its association with malaria severity is unknown.

The aim of this retrospective cohort study was to determine the prevalence of hyponatraemia and its association with malaria severity in a large cohort of patients with imported malaria caused by various *Plasmodium *species.

**Methods:**

All patients that were diagnosed with malaria in the Harbour Hospital and Institute for Tropical Diseases in Rotterdam in the period 1999-2009 and who had available serum sodium on admission were included. Severe malaria was defined according to the modified WHO criteria. Prevalence of hyponatraemia and its association with malaria severity were investigated by univariate comparison, ROC analysis and multivariate logistic regression analysis.

**Results:**

A total of 446 patients with malaria (severe falciparum malaria n = 35, non-severe falciparum malaria n = 280, non-falciparum malaria n = 131) was included. Hyponatraemia was present in 207 patients (46%). Prevalence and severity of hyponatraemia were greatest in severe falciparum malaria (77%, median serum sodium 129 mmol/L), followed by non-severe falciparum malaria (48%, median serum sodium 131 mmol/L), and non-falciparum malaria (34%, median serum sodium 132 mmol/L). Admission serum sodium < 133 mmol/L had a sensitivity of 0.69 and a specificity of 0.76 for predicting severe malaria. Multivariate logistic regression showed that serum sodium < 131 mmol/L was independently associated with severe falciparum malaria (odds ratio 10.4, 95% confidence interval 3.1-34.9). In patients with hyponatraemia, hypovolaemia did not appear to play a significant role in the development of hyponatraemia when prerenal azotaemia and haematocrit were considered as surrogate markers for hypovolaemia.

**Conclusions:**

Hyponatraemia is common in imported malaria and is associated with severe falciparum malaria. From a clinical point of view, the predictive power of hyponatraemia for severe malaria is limited. The precise pathophysiological mechanisms of hyponatraemia in malaria require further study.

## Background

Hyponatraemia has long been recognized as a complication of malaria [1]. The incidence of hyponatraemia in malaria has mostly been studied in endemic areas focusing on children with severe *Plasmodium falciparum *malaria and was approximately 55% [1-3]. Two studies in adults in Bangladesh and Thailand found incidences of 57% and 37%, respectively [4,5]. However, few studies have been performed in non-immune populations or in patients infected with other *Plasmodium *species.

The pathophysiology of hyponatraemia in malaria remains unclear but several studies have suggested that increased secretion of vasopressin, either appropriately or inappropriately, plays an important role [4,6-10]. Although a recent study suggested that the outcome of patients with malaria and hyponatraemia is good [4], cerebral oedema may still occur in rare cases [11].

The aim of this retrospective cohort study was to investigate the prevalence and severity of hyponatraemia in a large cohort of predominantly adult non-immune travellers with imported malaria caused by various *Plasmodium *species and its relationship with malaria severity.

## Methods

### Patients

The Harbour Hospital is a 161-bed general hospital located in Rotterdam, The Netherlands. It also harbours the Institute of Tropical Diseases, which serves as a national referral centre. All patients diagnosed with malaria in our hospital in the 10-year-period between January 1^st ^1999 and January 1^st ^2009 were included. Patients were identified by screening the malaria database of the Department of Parasitology. Of all patients thus identified, demographic, clinical and laboratory data were collected using a standardized form, and subsequently stored in an electronic database.

### Laboratory investigations

Available laboratory examinations included red and white blood cells, haematocrit, platelets, serum electrolytes, C-reactive protein (CRP), total bilirubin, serum creatinine and urea, liver enzymes, lactate dehydrogenase (LDH), blood glucose and plasma lactate. Serum sodium concentration was measured using indirect potentiometry (Beckman Synchron UniCel DxC 600 analyser). Blood smears (thin and thick films) were obtained from finger pricks and stained with Giemsa for parasite counts. Malaria was diagnosed by Quantitative Buffy Coat analysis, *P. falciparum *Histidine-Rich-Protein 2 screening (now ICT Malaria, Binax) and conventional microscopy with subsequent specification of the *Plasmodium *species. Multiple malaria episodes in a single patient were only regarded as separate cases if caused by true re-infection; recrudescent infections of *P. falciparum *and *Plasmodium malariae *and relapses of *Plasmodium vivax *and *Plasmodium. ovale *were excluded. Patients with a mixed infection of *P. falciparum *with another *Plasmodium *species were considered as having *P. falciparum *malaria.

### Definitions

*Severe malaria: *Patients were considered as having severe *P. falciparum *malaria if they met predefined modified World Health Organization (WHO) criteria for severe malaria on admission or during hospitalization [12] ("severity criteria"):

• A Glasgow Coma Scale (GCS) score < 11 (indicating cerebral malaria) *or*

• Anaemia (haematocrit < 0.20 L/L with parasite count > 100.000/μL) *or*

• Jaundice (serum bilirubin > 50 μmol/L with parasite count > 100.000/μL) *or*

• Renal impairment (urine output < 400 mL/24 h and serum creatinine > 250 μmol/L) *or*

• Hypoglycaemia (blood glucose < 2.2 mmol/L) *or*

• Hyperparasitaemia (> 10% parasitaemia) *or*

• Shock (systolic blood pressure < 80 mm Hg with cold extremities)

*Hyponatraemia: *Hyponatraemia was defined as a serum sodium concentration of less than 135 mmol/L.

### Statistical analysis

All data were not Normally distributed (Kolmogorov-Smirnov test) and are, therefore, presented as medians and range. Univariate comparisons were performed using the Kruskall-Wallis test (three groups) with Dunn's post-hoc tests, or the Mann-Whitney test (two groups). Correlations were analysed using Spearman rho (r_s_) and Wilcoxon signed rank test. The prognostic value of serum sodium for malaria severity was determined by a receiver operating characteristic (ROC) analysis. To analyse if hyponatraemia was also independently associated with malaria severity, a logistic regression analysis was performed using a backward stepwise conditional approach. In the latter analysis only patients with falciparum malaria were included (because only the falciparum species can cause severe malaria) and the presence of severe malaria was defined as the outcome.

## Results

### Patient characteristics

Serum sodium concentration on admission was available for 446 of the 477 malaria cases (93.5%), and they comprised the study population. Infection was most commonly acquired in Africa (75%) and Asia (14%). Infections with *P. falciparum *accounted for the majority of cases (n = 315, 70.6%). *P. falciparum *infection was classified as severe (n = 35) or non-severe (n = 280). One hundred and thirty-one patients had non-falciparum malaria, which consisted of *P. vivax *(n = 92, 70%,), *P. ovale *(n = 33, 25%) and *P. malariae *(n = 6, 5%). True re-infection occurred in 13 patients, and four patients had a mixed infection of *P. falciparum *and another *Plasmodium *species. Table 1 shows the comparison of the demographic characteristics, vital signs, and laboratory data on admission in the groups with severe and non-severe falciparum malaria, and non-falciparum malaria.

**Table 1:** Patient characteristics at initial presentation

	Severe *P. falciparum* (n = 35)	Non-severe *P. falciparum* (n = 280)	Non-falciparum (n = 131)	*P*-value*
**Demographics**				
Age, years	44 (4 - 70)	39 (4 - 78)	35.5 (8 - 77)	0.007^*B*^
Male, female, n (%)	21 (58), 14 (42)	202 (72), 78 (28)	87 (66), 44 (34)	N.S.
**Vital signs on admisson**				
Body temperature, °C	38.2 (36.2 - 41.2)	38.6 (35.7 - 41.0)	38.6 (35.0 - 41.5)	N.S.
Pulse rate, beats per minute	101 (50 - 150)	92 (45 - 150)	89 (58 - 138)	0.02^*A*^
Systolic blood pressure, mm Hg^†^	115 (80 - 160)	120 (80 - 90)	120 (70 - 196)	N.T.
GCS < 11, n (%)^†^	3 (9)	0 (0)	0 (0)	N.T.
**Laboratory data on admission**				
C-reactive protein, mg/L	184 (91 - 265)	77 (5 - 320)	71 (8 - 213)	< 0.0001^*A*^
Haematocrit, L/L^†^	0.35 (0.17 - 0.51)	0.40 (0.19 - 0.54)	0.38 (0.22 - 0.53)	N.T.
Leucocyte count, × 10^9^/L	7.1 (3.2 - 18.5)	4.9 (1.3 - 13.2)	5.3 (1.9 - 15.3)	< 0.0001^*A*^
Thrombocytes, × 10^9^/L	36 (3 - 188)	98 (11 - 433)	94 (10 - 292)	< 0.0001^*A*^
Serum sodium, mmol/L	130 (115 - 146)	135 (119 - 145)	136 (124 - 148)	< 0.0001^*A*^
Serum potassium, mmol/L	3.7 (2.7 - 4.7)	3.8 (2.5 - 5.1)	3.8 (2.7 - 5.4)	N.S.
Serum creatinine, μmol/L^†^	114 (48 - 871)	94 (41 - 228)	87 (46 - 477)	N.T.
Serum glucose, mmol/L	6.3 (4.1 - 12.5)	6.6 (4.4 - 33.8)	6.4 (4.7 - 22.1)	N.S.
Total bilirubin, μmol/L^†^	53 (15 - 416)	21 (4 - 262)	21.5 (3 - 91)	N.T.
Alanine-aminotransferase, U/L	58 (11 - 655)	35 (3 - 265)	31 (6 - 198)	0.001^*A*^
Lactate dehydrogenase, U/L	890 (308 - 4113)	455 (224 - 2724)	428 (185 - 1395)	< 0.0001^*A*^
Plasma lactate (mmol/L)	2,3 (1,2 - 5,8)	1,4 (0,5 - 4,4)	1,3 (0,7 - 3,0)	< 0.001^*B*^
Parasite load (trophozoites/μL)^†^	239,200 (520 - 1,110000)	5300 (2 - 385,000)	N/A	N.T.
**Duration hospitalisation**, days	8 (3 - 19)	5 (1 - 16)	2 (1 - 11)	< 0.0001^*A+C*^

* Using Kruskall-Wallis and Dunn's post-hoc tests. Parameters that are included in the modified WHO severity criteria for severe falciparum malaria (indicated by † [12]) were not tested (N.T.). N.S. denotes 'not significant'.

*A *group 1 vs. group 3; *B *group 1 vs. groups 2 and 3; *C *group 2 vs. group 3

### Characteristics of patients with severe malaria

Thirty-two patients fulfilled one or more of the severity criteria at initial presentation (GCS < 11 n = 3; anaemia with a parasite count exceeding 100,000 trophozoites per μL n = 3; icterus with a parasite count exceeding 100,000 trophozoites per μL n = 17; acute oliguric renal insufficiency n = 4; hypoglycaemia n = 0; hyperparasitaemia n = 12 and shock n = 2, respectively). Three patients did not fulfil the criteria for severe disease on admission but their clinical course deteriorated shortly hereafter. Admission plasma lactate levels were significantly increased in patients with severe malaria as compared with patients with uncomplicated falciparum malaria and patients with non-falciparum malaria (table 1). Eventually, at admission to the intensive care unit (ICU), all 35 patients fulfilled one or more of the severity criteria (cerebral malaria or impaired conscious level n = 9; anaemia with a parasite count exceeding 100,000 trophozoites per μL n = 3; icterus with a parasite count exceeding 100,000 trophozoites per μL n = 19; acute oliguric renal insufficiency n = 5; shock n = 2, respectively). The first arterial blood gas analysis on ICU showed a median bicarbonate level of 21 mmol/L (range 17 to 26 mmol/L) and a median base deficit of 2 (range -3 to 9). Two patients needed mechanical ventilation, whereas three patients needed haemodialysis. Twenty-four patients received exchange transfusion. Details of this standardized adjunct treatment have been published elsewhere [13]. One patient with severe falciparum malaria died of cerebral malaria.

### Hyponatraemia in imported malaria

**Figure 1:**
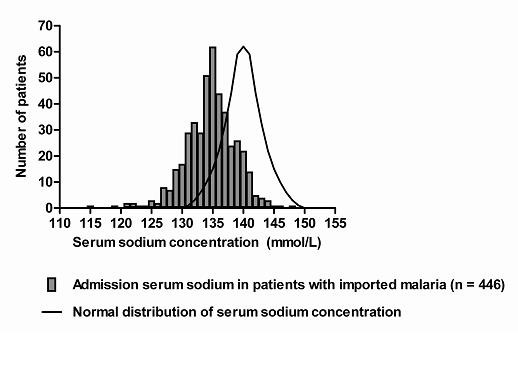
**Distribution of serum sodium in patients with imported malaria**.

**Figure 2:**
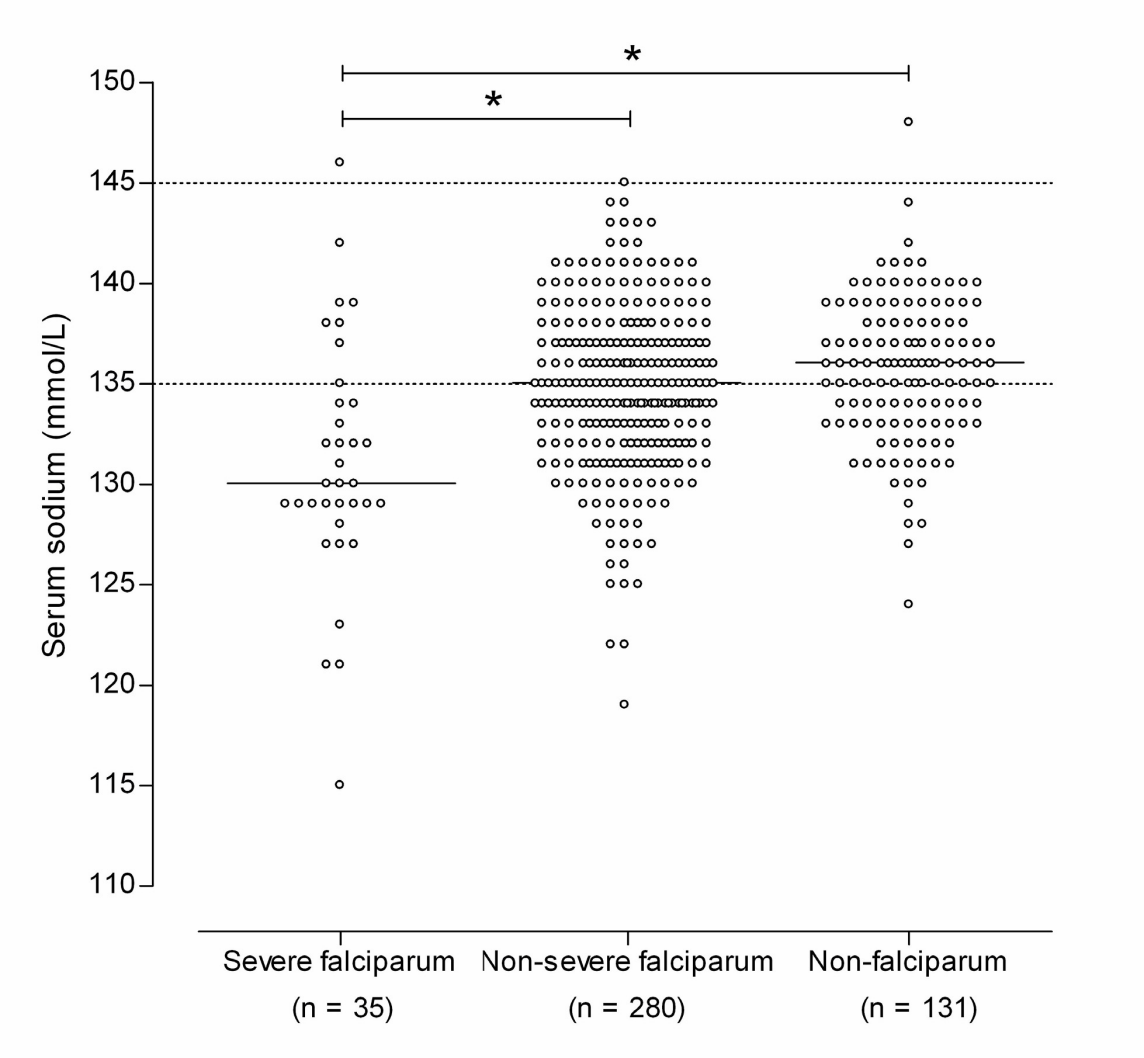
**Distribution of serum sodium in malaria patients grouped by causative Plasmodium species**. Hyponatraemia was present in 27/35 (77%) of patients with severe *P. falciparum *infection, in 135/280 (48%) of patients with non-severe *P. falciparum *infection, and in 45/131 (34%) of patients with non-falciparum infection. Dashed lines indicate normal reference range. Bars indicate the median.

Hyponatraemia was present in 207 patients (46%). The distribution of serum sodium in the whole population seemed to be shifted to lower values (Figure 1). Figure 2 shows the distribution of serum sodium in patients with severe *P. falciparum*, non-severe *P. falciparum*, and non-falciparum malaria. Only two patients (0.4%) had a serum sodium concentration exceeding 145 mmol/L. When only patients with hyponatraemia were considered, hyponatraemia was more severe in severe falciparum malaria (median serum sodium 129 mmol/L) compared with both non-severe falciparum and non-falciparum malaria (median serum sodium 131 and 132 mmol/L, respectively, p < 0.05). In the whole population, prerenal azotaemia (defined as a serum urea: creatinine ratio > 1: 10) was not more common (11/207 or 5.3% vs. 7/239 or 2.9%, p = 0.2) and haematocrit values (median 0.39 vs. 0.39, p = 0.6) were not higher in patients with hyponatraemia and malaria. Other causes of hyponatraemia appeared rare. Only three patients with malaria and hyponatraemia also used drugs associated with hyponatraemia (1 thiazide diuretic, 1 several diuretics, 1 risperidone) [14]. None of the patients had severe heart or liver failure, and none of the patients had known pre-existing hyponatraemia. A significant correlation was demonstrated between sodium levels and CRP levels on admission for all patients with imported malaria (n = 428; r_s _= -0.36; p < 0.0001) but also for patients with infections solely caused by *Plasmodium falciparum *(n = 298; r_s _= -0.41; p < 0.0001).

### Hyponatraemia and malaria severity

**Figure 3:**
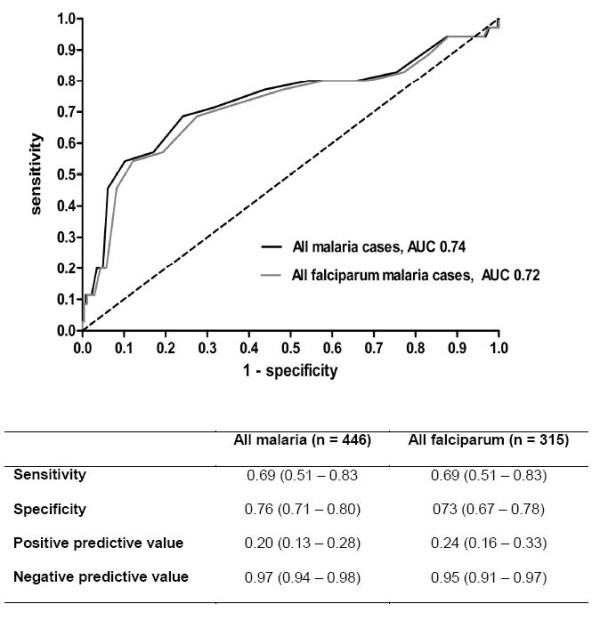
**Receiver operating characteristics curve of serum sodium concentration for malaria severity**.

The area-under the ROC-curve for the predictive value of serum sodium concentration for malaria severity was 0.72 for *P. falciparum *infections and 0.74 for all patients with malaria (Figure 3). A cut-off of 133 mmol/L was identified as having the best discriminatory performance (sensitivity 0.69, specificity 0.76, positive predictive value 0.20, negative predictive value 0.97). Variables significant on univariate analysis that were not severity criteria (Table 1) were entered as dichotomous variables in the multivariate model using different cut-off points. The following parameters were independently associated with severe falciparum malaria: serum sodium < 131 mmol/L, CRP > 175 mg/L, LDH > 750 U/L, thrombocytes < 20 × 10^9^/L and leukocytes > 6.5 × 10^9^/L (Table 2). When the severity criteria were also entered in the model, only serum sodium < 131 mmol/L and CRP > 175 mg/L remained independent predictors of severe falciparum malaria (both p < 0.05).

**Table 2:** Logistic regression analysis showing independent predictors of severe falciparum malaria

Variable	β	*SE *β	Wald's χ	*df*	*P*-value	Odds ratio (95% CI)
Constant	-6.1	1.32	21,400	1	< 0.001	N/A
Serum sodium < 131 mmol/L	2.3	0.62	14,286	1	< 0.001	10.4 (3.1 - 34.9)
C-reactive protein > 175 mg/L	1.6	0.58	7,472	1	0.006	4.8 (1.6 - 15.0)
Lactate dehydrogenase > 750 U/L	1.8	0.58	9,288	1	0.002	5.9 (1.9 - 18.7)
Thrombocytes < 20 × 10^9^/L	3.7	1.03	12,761	1	< 0.001	40.0 (5.3 (302.8)
Leukocytes > 6.5 × 10^9^/L	2.3	0.60	14,384	1	< 0.001	9.7 (3.0 - 31.5)

### Clinical course and outcome

The median duration of hospital stay is shown in Table 1. Three of the 35 patients with severe falciparum malaria met the criteria for severe malaria only hours after admission when their parasite counts increased to above cut-off levels or their clinical course deteriorated. Interestingly, two of them were hyponatraemic on admission (serum sodium 130 and 132 mmol/L). Follow-up serum sodium concentrations were available for 24 of 27 patients with severe falciparum infection and hyponatraemia. Serum sodium normalized within 24 hours in seven patients. It took between 24 and 96 hours in seven patients, and more than 96 hours in eight patients. In three patients the time in which serum sodium normalized was unknown. Patients with more severe hyponatraemia on admission remained hyponatraemic for a longer period of time (p < 0.01). There was no significant correlation between sodium levels and GCS in patients with severe malaria (n = 35; r_s _= -0.036; p = 0.84).

## Discussion

The present study shows that hyponatraemia is common in patients with imported malaria, and is associated with severe disease in *P. falciparum *malaria. The incidence of hyponatraemia in this study (77% severe *P. falciparum*, 48% non-severe *P. falciparum*, and 34% non-falciparum) is comparable to that found in other studies. Holst *et al *[7] found hyponatraemia in 13 of 17 (76%) non-immune travellers with severe *P. falciparum *infection, whereas Kockaerts *et al *[15] reported an incidence of 53% in a cohort of 101 patients with imported malaria, although no differentiation according to Plasmodium species or severity of malaria was made. In children with imported malaria, lower incidences were found (5/20 or 25% by Viani *et al *[16], 16/192 or 8% by Ladhani *et al *[2]).

Interestingly, in the present study, a serum sodium < 131 mmol/L, CRP > 175 mg/L, LDH > 750 IU/L, thrombocyte count < 20 × 10^9^/L, and leukocyte count > 6.5 × 10^9^/L were all independently associated with severe falciparum malaria (as defined by the modified WHO severity criteria [12]). Some of these parameters like thrombocytopaenia [17] and leukocytosis [18] have been reported as risk factors for severe malaria or a fatal outcome but this has not been a universal finding [19]. When the severity criteria were also entered in the regression model, serum sodium < 131 mmol/L and CRP > 175 mg/L were the only remaining independent variables with Odds ratio's of 10.4 (95% Confidence Interval 3.1 - 34.9) and 4.8 (95% CI 1.6 - 15.0) for severe malaria, respectively, suggesting that determination of these parameters might contribute to an early recognition of patients with severe malaria on admission. However, there are some important drawbacks, which may limit its usefulness in clinical practice. First, sodium had a poor positive predictive value (0.20) for severe malaria. The observations that hyponatraemia may be seen in imported malaria caused by any Plasmodium species as well as in other infectious diseases suggest that hyponatraemia *per se *is unlikely to represent an exclusive feature of falciparum malaria but merely reflects the effects of severity of any disease rather than malaria itself. Second, hyponatraemia is a well recognized indicator of disease severity and predictor of mortality regardless of its cause, as has been observed in several studies with hospitalized adult patients [20,21]. As such, the recognition that lower sodium occurs more frequently in severe malaria will probably not significantly change the monitoring and management of the patient since most physicians would already have a higher index of suspicion for a complicated course. Third, the finding that sodium is an independent risk factor for severe malaria contrasts with the findings of two recent studies. In the first study [22], performed in several malaria-endemic countries, involving more than 1000 adults individuals with severe malaria (defined by the same modified WHO criteria) only base deficit and cerebral malaria (measured with GCS) but not sodium were found to be main independent predictors of outcome. Moreover, in the other study [23] involving 482 individuals with imported falciparum malaria, sodium was not identified as an independent risk factor for severe falciparum malaria. The reason for this discrepancy with the present findings is not immediately apparent but might relate to differences in the number of elderly patients [24] and differences in ethnicity [23]. For example, of the 482 individuals with imported malaria [23], 68% of the patients were of black ethnicity, which was associated with a reduced risk of developing WHO-defined severe falciparum malaria, whereas white patients had odds that were 8-fold higher. In the present study patients of black ethnicity constituted only a minority of the patients. Fourth and probably most important from a clinical point of view, other parameters like coma and parameters of impaired tissue perfusion (such as acidosis [25], elevated lactate level [25,26] or base deficit [22]) have been reported as more powerful risk factors for severe malaria or a fatal outcome than sodium.

Even though the predictive power of hyponatraemia for severe malaria may be limited in clinical practice, its pathophysiology is certainly puzzling. Suggested mechanisms are absolute sodium deficit due to cerebral salt wasting, excessive sweating or loss in the gastrointestinal or urinary tract [3], and the secretion of vasopressin which can be either appropriate, in case of volume depletion [4], or inappropriate as in the syndrome of inappropriate antidiuretic hormone secretion [10] or reset osmostat [8]. There is, however, no consensus as to their relative contributions Although it has been suggested that hyponatraemia is associated with worse outcome and should be corrected [27], a recent study found that hyponatraemia is associated with preserved consciousness and even a decreased mortality in severe *P. falciparum *malaria [4]. In the setting of hypovolaemia, the hyponatraemia was likely related to a continued oral hypotonic fluid intake in those patients who had preserved consciousness and the hyponatraemia required no therapy beyond rehydration given the observation that plasma sodium normalized with crystalloid rehydration within 24 hours after admission [4]. Although the present study was not designed to investigate the pathophysiology of hyponatraemia in malaria, a number of observations might argue against a role for hypovolaemia in the pathophysiology of hyponatraemia in the present study. When prerenal azotaemia and haematocrit were considered as surrogate markers for hypovolaemia, prerenal azotaemia was not more common and haematocrit was not higher in hyponatraemic patients as compared with normonatraemic individuals. In addition, hyponatraemia persisted for days in one third of the patients despite rapid volume resuscitation with normal saline. An explanation for this difference could be that the patients described by Hanson *et al *[4] presented late to hospital and were more ill on admission as evidenced by the high number of comatose patients and the higher mortality rate. The inverse relationship between plasma sodium and GCS could not be reproduced in the present study but might be underpowered by the relatively low number of comatose patients. Although speculative, an other mechanism may have contributed to hyponatraemia in the present study. It is certainly interesting to note that the pro-inflammatory cytokine interleukin-6 is elevated in malaria and also implicated in the non-osmotic release of vasopressin [28,29]. The delayed normalization of serum sodium concentration as was observed in the present study, might reflect the persistent elevation of inflammatory cytokines, which are known to remain increased for several weeks in some patients with severe malaria [30]. Previously, a relationship between the development of in-hospital hyponatraemia and a rise in CRP was demonstrated, which is not only another illustration of this mechanism [20] but also in line with the observed correlation between sodium levels and CRP levels in patients with imported malaria in the present study. Additional studies are needed to further unravel the intriguing pathophysiology of hyponatraemia in malaria.

In conclusion, hyponatraemia is common in imported malaria and associated with severe falciparum malaria. From a clinical point of view, however, the predictive power of hyponatraemia for severe malaria is limited. The precise pathophysiological mechanisms of hyponatraemia in malaria require further study.

## Competing interests

The authors declare that they have no competing interests.

## Authors' contributions

MEvW, DAH and EJH performed the data analysis and wrote the first draft of the manuscript. PJJvG and MEvW contributed to the data acquisition. RZ and PJJvG contributed to the data analysis and made substantial changes to the manuscript. All authors have seen and approved the final version.

## References

[B1] English MC Waruiru C Lightowler C Murphy SA Kirigha G Marsh K Hyponatraemia and dehydration in severe malaria Arch Dis Child 1996 74 201 205 10.1136/adc.74.3.201 8787422 PMC1511412

[B2] Ladhani S Patel VS El Bashir H Shingadia D Changes in laboratory features of 192 children with imported falciparum malaria treated with quinine Pediatr Infect Dis J 2005 24 1017 1020 10.1097/01.inf.0000183774.22593.7c 16282945

[B3] Sowunmi A Hyponatraemia in severe falciparum malaria: a clinical study of nineteen comatose African children Afr J Med Med Sci 1996 25 47 52 9110054

[B4] Hanson J Hossain A Charunwatthana P Hassan MU Davis TM Lam SW Chubb SA Maude RJ Yunus EB Haque G White NJ Day NP Dondorp AM Hyponatremia in severe malaria: evidence for an appropriate anti-diuretic hormone response to hypovolemia Am J Trop Med Hyg 2009 80 141 145 19141852 10.4269/ajtmh.2009.08-0393 PMC2843441

[B5] Thanachartwet V Krudsood S Tangpukdee N Phumratanaprapin W Silachamroon U Leowattana W Wilairatana P Brittenham GM Looareesuwan S Neild GH Hyponatraemia and hypokalaemia in adults with uncomplicated malaria in Thailand Trop Doct 2008 38 155 157 10.1258/td.2007.070112 18628541 PMC3123524

[B6] Fryatt RJ Teng JD Harries AD Moody AH Hall AP Forsling ML Plasma and urine electrolyte concentrations and vasopressin levels in patients admitted to hospital for falciparum malaria Trop Geogr Med 1989 41 57 60 2669290

[B7] Holst FG Hemmer CJ Kern P Dietrich M Inappropriate secretion of antidiuretic hormone and hyponatremia in severe falciparum malaria Am J Trop Med Hyg 1994 50 602 607 8203710 10.4269/ajtmh.1994.50.602

[B8] Miller LH Makaranond P Sitprija V Suebsanguan C Canfield CJ Hyponatraemia in malaria Ann Trop Med Parasitol 1967 61 265 79 4867548 10.1080/00034983.1967.11686487

[B9] Sitprija V Napathorn S Laorpatanaskul S Suithichaiyakul T Moollaor P Suwangool P Sridama V Thamaree S Tankeyoon M Renal and systemic hemodynamics, in falciparum malaria Am J Nephrol 1996 16 513 519 10.1159/000169042 8955763

[B10] Sowunmi A Newton CR Waruiru C Lightman S Dunger DB Arginine vasopressin secretion in Kenyan children with severe malaria J Trop Pediatr 2000 46 195 199 10.1093/tropej/46.4.195 10996978

[B11] Ustianowski A Schwab U Pasvol G Case report: severe acute symptomatic hyponatraemia in falciparum malaria Trans R Soc Trop Med Hyg 2002 96 647 648 10.1016/S0035-9203(02)90341-X 12625142

[B12] Tran TH Day NP Nguyen HP Nguyen TH Tran TH Pham PL Dinh XS Ly VC Ha V Waller D Peto TE White NJ A controlled trial of artemether or quinine in Vietnamese adults with severe falciparum malaria N Engl J Med 1996 335 76 83 10.1056/NEJM199607113350202 8649493

[B13] van Genderen PJJ Hesselink DA Bezemer JM Wismans PJ Overbosch D Efficacy and safety of exchange transfusion as an adjunct therapy for severe *Plasmodium falciparum *malaria in nonimmune travellers: a 10-year single-center experience with a standardized treatment protocol Transfusion 2010 50 787 794 10.1111/j.1537-2995.2009.02488.x 19951317

[B14] Liamis G Milionis H Elisaf M A review of drug-induced hyponatremia Am J Kidney Dis 2008 52 144 153 10.1053/j.ajkd.2008.03.004 18468754

[B15] Kockaerts Y Vanhees S Knockaert DC Verhaegen J Lontie M Peetermans WE Imported malaria in the 1990s: a review of 101 patients Eur J Emerg Med 2001 8 287 290 10.1097/00063110-200112000-00007 11785595

[B16] Viani RM Bromberg K Pediatric imported malaria in New York: delayed diagnosis Clin Pediatr (Phila) 1999 38 333 337 10.1177/000992289903800603 10378090

[B17] Gerardin P Rogier C Ka AS Jouvencel P Brousse V Imbert P Prognostic value of thrombocytopenia in African children with falciparum malaria Am J Trop Med Hyg 2002 66 686 691 12224575 10.4269/ajtmh.2002.66.686

[B18] Ladhani S Lowe B Cole AO Kowuondo K Newton CR Changes in white blood cells and platelets in children with falciparum malaria: relationship to disease outcome Br J Haematol 2002 119 839 847 10.1046/j.1365-2141.2002.03904.x 12437669

[B19] Moulin F Lesage F Legros AH Maroga C Moussavou A Guyon P Marc E Gendrel D Thrombocytopenia and *Plasmodium falciparum *malaria in children with different exposures Arch Dis Child 2003 88 540 541 10.1136/adc.88.6.540 12765928 PMC1763122

[B20] Beukhof CM Hoorn EJ Lindemans J Zietse R Novel risk factors for hospital-acquired hyponatraemia: a matched case-control study Clin Endocrinol (Oxf) 2007 66 367 372 10.1111/j.1365-2265.2007.02741.x 17302870

[B21] Waikar SS Mount DB Curhan GC Mortality after hospitalization with mild, moderate, and severe hyponatremia Am J Med 2009 122 857 865 10.1016/j.amjmed.2009.01.027 19699382 PMC3033702

[B22] Hanson J Lee SJ Mohanty S Faiz MA Anstey NM Charunwatthana P Yunus EB Mishra SK Tjitra E Price RN Rahman R Nosten F Htut Y Hoque G Chau TTH Phu NHm Hien TT White NJ Day NPJ Dondorp AM A simple score to predict the outcome of severe malaria in adults Clin Infect Dis 2010 50 679 685 10.1086/649928 20105074 PMC4313369

[B23] Philips A Bassett P Zeki S Newman S Pasvol G Risk factors for severe disease in adults with falciparum malaria Clin Infect Dis 2009 48 871 878 10.1086/597258 19243243

[B24] Dondorp AM Lee SJ Faiz MA Mishra S Price R Tjitra E Than M Htut Y Mohanty S Yunus EB Rahman R Nosten F Anstey NM Day NP White NJ The relationship between age and the manifestations of and mortality associated with severe malaria Clin Infect Dis 2008 47 151 157 10.1086/589287 18533842

[B25] Day NP Phu NH Mai NT Chau TT Loc PP Chuong LV Sinh DX Holloway P Hien TT White NJ The pathophysiologic and prognostic significance of acidosis in severe adult malaria Crit Care Med 2000 28 1833 1840 10.1097/00003246-200006000-00025 10890629

[B26] Van Genderen PJ Meer IM van der Consten J Petit PLC van Gool T Overbosch D Evaluation of plasma lactate as a parameter for disease severity on admission in travelers with *Plasmodium falciparum *malaria J Travel Med 2005 12 261 264 16256049 10.2310/7060.2005.12504

[B27] Enwere GC Ota MO Obaro SK Electrolyte derangement in cerebral malaria: a case for a more aggressive approach to the management of hyponatraemia Ann Trop Med Parasitol 2000 94 541 547 11064755 10.1080/00034983.2000.11813576

[B28] Mastorakos G Weber JS Magiakou MA Gunn H Chrousos GP: Hypothalamic-pituitary-adrenal axis activation and stimulation of systemic vasopressin secretion by recombinant interleukin-6 in humans: potential implications for the syndrome of inappropriate vasopressin secretion J Clin Endocrinol Metab 1994 79 934 939 10.1210/jc.79.4.934 7962300

[B29] Palin K Moreau ML Sauvant J Orcel H Nadjar A Duvoid-Guillou A Dudit J Rabié A Moos F Interleukin-6 activates arginine vasopressin neurons in the supraoptic nucleus during immune challenge in rats Am J Physiol Endocrinol Metab 2009 296 e1289 1299 10.1152/ajpendo.90489.2008 19258490

[B30] Ballal A Saeed A Rouina P Jelkmann W Effects of chloroquine treatment on circulating erythropoietin and inflammatory cytokines in acute *Plasmodium falciparum *malaria Ann Hematol 2009 88 411 415 10.1007/s00277-008-0636-z 19031076

